# Assessing the impoverishing effects, and factors associated with the incidence of catastrophic health care payments in Kenya

**DOI:** 10.1186/s12939-017-0526-x

**Published:** 2017-02-06

**Authors:** Edwine W. Barasa, Thomas Maina, Nirmala Ravishankar

**Affiliations:** 1 Health Economics Research Unit, KEMRI Wellcome Trust Research Programme, P.O Box 43640-00100, Nairobi, Kenya; 2 Nuffield department of Medicine, University of Oxford, Oxford, UK; 3 USAID Health Policy Plus Project, Palladium group, 1331 Pennsylvania Ave NW, Suite 600, Washington, DC 20004 USA; 4 Independent consultant, Nairobi, Kenya

**Keywords:** Household health spending, Out of pocket health spending, Catastrophic health spending, Financial risk protection, Kenya

## Abstract

**Background:**

Monitoring the incidence and intensity of catastrophic health expenditure, as well as the impoverishing effects of out of pocket costs to access healthcare, is a key part of benchmarking Kenya’s progress towards reducing the financial burden that households experience when accessing healthcare.

**Methods:**

The study relies on data from the nationally-representative Kenya Household Expenditure and Utilization Survey conducted in 2013 (*n* =33,675). We undertook health equity analysis to estimate the incidence and intensity of catastrophic expenditure. Households were considered to have incurred catastrophic expenditures if their annual out of-pocket health expenditures exceeded 40% of their annual non-food expenditure. We assessed the impoverishing effects of out of pocket payments using the Kenya national poverty line. We distinguished between direct payments for healthcare such as payments for consultation, medicines, medical procedures, and total healthcare expenditure that includes direct healthcare payments and the cost of transportation to and from health facilities. We used logistic regression analysis to explore the factors associated with the incidence of catastrophic expenditures.

**Results:**

When only direct payments to healthcare providers were considered, the incidence of catastrophic expenditures was 4.52%. When transport costs are included, the incidence of catastrophic expenditure increased to 6.58%. 453,470 Kenyans are pushed into poverty annually as a result of direct payments for healthcare. When the cost of transport is included, that number increases by more than one third to 619,541. Unemployment of the household head, presence of an elderly person, a person with a chronic ailment, a large household size, lower household social-economic status, and residence in marginalized regions of the country are significantly associated with increased odds of incurring catastrophic expenditures.

**Conclusions:**

Kenyan policy makers should prioritize extending pre-payment mechanisms to more vulnerable groups, specifically the poor, the elderly, those suffering from chronic ailments and those living in marginalized regions of the country. The range of services covered under these mechanisms should also be extended such that the proportion of direct costs paid to access care is reduced. Policy makers should also prioritize reducing supply side bottlenecks such as availability of healthcare facilities in close proximity to the population, especially in rural and marginalized areas, and improvements in quality of care. For the poor and the vulnerable, initiatives to cover the cost of transport to and from a health facility, such as transport vouchers could also be explored.

## Background

There is increasing commitment by low and middle income countries (LMICs) to achieve universal health coverage (UHC) [1], the goal of which is to ensure that everyone has access to needed healthcare services without getting into financial ruin or impoverishment [2]. This commitment has culminated in the inclusion of UHC in the Sustainable Development Goals (SDGs), which were adopted by world leaders in 2015 to articulate global development priorities until 2030 [3]. Health systems in LMICs are still heavily dependent on people making out-of-pocket (OOP) payments to cover the costs of healthcare at the time when they use the services [4, 5]. Over 100 million people globally are pushed into poverty annually as a result of OOP healthcare payments [6]. Ensuring that households are protected from such catastrophic expenditure – also referred to as financial risk protection - is recognized as a desirable health policy objective [7–10]. Therefore, tracking the extent of financial risk protection achieved by different countries has been proposed as a key part of the SDG monitoring framework [11].

Kenya’s health sector is financed by a mix of public, private, and donor resources. Between 2009 and 2013, donor financing reduced from 34.5 to 25.6%, while financing from public sources increased from 28.8 to 33.5% (Table 1) [12]. Private financing for health increased from 36.7 to 39.8% over the same time period. This is worrying because a huge proportion of private funding is in the form of out of pocket (OOP) payments. Specifically, OOP spending as a proportion of total health expenditure (THE) increased from 25% in 2009 to 29% in 2013.

**Table 1:** Selected health financing indicators for Kenya

	Year	
Indicator	2007	2013
Proportion of Kenyans covered by health insurance [17]	10.0%	17.1%
Financing sources as a percentage of total health expenditure (THE) [62]	2009	2013
Percentage of THE financed by public sources	28.8%	33.5%
Percentage of THE financed by donors	34.5%	25.6%
Percentage of THE financed by private sources	36.7%	39.8%
Percentage of THE paid for through Out-of-pocket expenditure	25%	29%
Total health expenditure (THE) per capita (USD)	55.8	66.6
THE as a percentage of gross domestic product (GDP)	5.4%	6.8%
Government health expenditure as a percentage of total government expenditure	4.6%	6.1%
Public expenditure on health as a percentage of GDP	1.6%	2.3%

The Kenyan government has over the years undertaken a number of health system reforms that have had an impact on the extent to which the population has financial risk protection. After independence in 1963, the country abolished user fees that had been imposed on health services at public facilities by the colonial government [13]. The Kenyan health sector was predominantly tax funded until 1989, at which point the country introduced user fees in public hospitals and peripheral health facilities (health centers and dispensaries) that offer outpatient primary healthcare services [13, 14]. However, due to social justice concerns, user fees were abolished in 1990, but reintroduced again in 1992 because of budgetary constraints [14, 15]. In 2004, the Kenyan government abolished user fees in public health centers and dispensaries, except for a flat registration fee of Kenyan shillings (KES) 10 in dispensaries and KES 20 in health centers, which translates to US dollar (USD) 0.1 and USD 0.2 respectively [15]. Public hospitals were however allowed to continue collecting user fees under a cost-sharing arrangement where hospitals received partial supply side subsidies from the central government, and charged fees to users of healthcare services. In 2013, after the election of a new government, user fees were completely abolished in health centers and dispensaries [16].

Despite the abolition of user fees at public health centers and dispensaries, OOP payments continue to be a problem in the Kenyan health system. A number of factors could explain this. First, services at public hospitals (which still operate under the cost-sharing policy) as well as all levels of private healthcare facilities are still paid for through OOP payments. Second, health insurance coverage in Kenya remains low even though it has increased from 10 to 17.1% between 2007 and 2013 [17]. Of those covered by health insurance, 99% are covered by the National Hospital Insurance Fund (NHIF), a state entity with the mandate to provide social health insurance, while the remaining 1% is covered by private and community based health insurance [17]. However, health insurance mobilizes only 5% of current health expenditure in Kenya, implying that the depth of cover is low, and hence necessitating OOP (Table 1). Further, the health sector continues to be under-prioritized by the government. While the Abuja declaration recommends that governments allocate at least 15% of their budgets to the health sector [18], Kenya allocated 6.1%. Further, while it has been recommended that, for countries to accelerate progress towards achieving UHC, government’s expenditure on health should at least be 5% of their GDP [19], Kenya’s share was 2.1% in 2013.

OOP payments deter some Kenyans from seeking care and cause others to become impoverished as a result of paying for care. A previous study by Chuma and colleagues estimated that 14.8% of households experienced catastrophic healthcare expenditure in 2007 [20]. Further it was estimated that nearly 1.5 million Kenyans were pushed into poverty due to catastrophic health spending [20]. In this paper we present an analysis of catastrophic costs and impoverishment using the most recent data from the 2013 Kenya Household Expenditure and Utilization Survey (KHHEUS). The objectives of this study are to 1) examine the incidence and intensity of catastrophic health expenditures, 2) to examine the impoverishing effect of OOP health spending, and, 3) to explore factors that are associated with catastrophic health spending in Kenya.

Our analysis contributes to the policy dialogue around UHC in Kenya and as well as the broader literature on catastrophic health spending in three ways. First, by using recent data, it provides policy makers with information to take stock of progress (or lack thereof) on improving financial risk protection among the population, and provides a benchmark for the future. Second, unlike the analysis by Chuma and colleagues, and most analyses that only consider catastrophe and impoverishment due to direct payments made to healthcare providers, we also consider the impact of transport costs borne by users to access healthcare services. This is based on the recognition that transport costs are often quite significant when compared to direct payments to healthcare providers [9]. Third, unlike the analysis by Chuma and colleagues, we explore the association between catastrophic health spending and a range of individual, household, and county-level covariates. Literature is scarce on factors associated with catastrophic healthcare expenditures [21]. Identifying these relationships provides policy levers that can be targeted by decision makers interested in intervening to improve financial risk protection in Kenya [21].

## Methods

### Study setting

Kenya is a lower middle income country in East Africa with a per capita Gross Domestic Product (GDP) of USD 1245. It has an estimated population of 44.86 million [22], 45.9% of which are estimated to be living below the national poverty line [23]. Kenya’s population is predominantly rural, with an estimated 65% of the population living in rural areas [17]. The country operates under a devolved system with two levels of governance: the national government and 47 semi-autonomous devolved units called county governments [24]. The Kenyan healthcare delivery system is pluralistic with a 50–50% split between public and private healthcare provision. Healthcare providers are organized into four tiers, namely community, primary care, county referral and national referral [25, 26]. Community health services include all community-based demand creation activities that are guided by the Ministry of Health (MOH) community strategy [25, 26]. Primary healthcare include services provided by public and private maternity homes, health centers and dispensaries. County referral services include first level referral hospitals that are managed by a given county. National referral services are comprised of national level facilities, where tertiary referral services are provided.

### Data sources

This analysis is based on the 2013 KHHEUS [17]. The KHHEUS collects detailed data on socio-demographic characteristics of households and individuals, household expenditures, healthcare spending, and individual level data on outpatient attendance (over a 4 week recall period) and inpatient hospitalization (over a 12 month recall period). The KHHEUS sample was drawn from the master sample developed and maintained by the Kenya National Bureau of Statistics (KNBS) based on a multistage sampling design [17]. The Kenya KHHEUS 2013 has a sample of 33,675 households drawn from 1347 selected clusters comprised of 533 (40%) urban and 814 (60%) rural clusters. The sample did not include institutionalized individuals. This sample was drawn from 44 out of the 47 counties in Kenya, because three counties, Mandera, Wajir, and Garissa, were not included in the survey. This was because the sampling clusters had not been updated in the KNBS master sample [17]. However, the sample was constructed to allow for representativeness at both the national and county levels as well as urban and rural domains.

Of the individuals sampled, 19.3% reported illness in the four weeks preceding the survey [17]. Of these, 80% sought care; care was forgone by 20% of individuals that had reported illness in the four weeks preceding the survey for a number of reasons that included 1) prohibitive costs of care 2) long distances to health facilities, and 3) illness not considered serious enough [17].

### Data analysis

#### Measuring the incidence and intensity of catastrophic spending

There is no consensus on the threshold for catastrophic health payments. In this analysis, households were considered to have incurred catastrophic expenditures if their total out of-pocket health costs in a year exceeded 40% of their annual non-food expenditure (referred to as capacity to pay) [9]. We chose this threshold given that it considers the effective income remaining after basic subsistence needs have been met, rather than the total household expenditure, and therefore represents the true capacity to pay for healthcare expenditure. In this paper, we analyzed the incidence of catastrophe due to direct healthcare payments (i.e. payments made to healthcare providers for services received) and also due to healthcare and transport payments (i.e. transport costs incurred for a return trip to and from a healthcare facility). To obtain healthcare OOP spending, we subtracted costs that were covered by health insurance (or other payment mechanisms such as borrowing without the expectation of paying back) from the total reported healthcare cost. Since the survey used a 4 week recall period for outpatient services, we annualized the outpatient OOP by multiplying them by 13. We classified households into wealth quintiles by using per capita consumption expenditure.

The catastrophic head count (HC), which represents the incidence of catastrophe, refers to the proportion of households that incurred catastrophic health payments and is estimated as follows [27]:

1 $$ \mathrm{H}\mathrm{C}={\displaystyle {\sum}_{i=1}^N}\ {E}_i $$

Where N is the sample size while E is an indicator that assumes a value of 1 if the OOP payments of a household i exceed the defined catastrophic expenditure threshold, and 0 if it does not. While catastrophic head count estimates the fraction of households that incur catastrophic costs, it does not give information on the extent of catastrophe (by how much a households OOP payment exceeds the catastrophic threshold). The catastrophic overshoot (O) provides this information. Catastrophic overshoot refers to the average degree by which OOP payments, as a proportion of total expenditure, exceeds the catastrophic payment threshold (Z). Catastrophic overshoot (O) is estimated as follows [27]:

2 $$ {0}_i={Q}_i\left(\frac{T_i}{X_i}- Z\right) $$

Where T_i_ represents the OOP payments of household i, X_i_ represents the household non-food consumption expenditure of household i, and Z represents the catastrophic threshold. Subsequently, the average catastrophic overshoot can be estimated as follows [27]:

3 $$ \mathrm{O}=\frac{1}{N}{\displaystyle {\sum}_{i=1}^N} O i $$

The intensity of catastrophic expenditure is computed by averaging the catastrophic overshoot over all households that exceed the catastrophic threshold. This measure, referred to as the mean positive overshoot (MPO), is computed as follows:

4 $$ \mathrm{M}\mathrm{P}\mathrm{O}=\frac{O}{HC} $$

#### Distributional profile of catastrophic incidence and intensity

The distributional profile of the incidence and intensity of catastrophic payments was assessed by computing concentration indices. The concentration index is derived by computing the covariance between the fractional rank of the individual sorted by socio-economic status and the variable of interest [27]. The index assumes a value ranging from −1 to +1. A negative concentration index indicates that the variable of interest is concentrated among the poor and vice versa [28]. The “traditional” concentration index is only appropriate when the variable of interest is measured on a ratio-scale [27]. Applying the “traditional” concentration index to binary (and bounded) variables, such as the incidence of catastrophic expenditure, is therefore problematic [29–31]. Wagstaff [32] and Erreygers [31] have proposed corrected concentration indices. While there is no consensus on which corrected concentration index is superior to the other [30, 33, 34], we chose to compute Erreygers concentration index [31]. Erreygers concentration index *E*(*h*) is computed as follows [31]:

5 $$ E(h) = \frac{8}{n^2\left({b}_h-{a}_h\right)}{\displaystyle {\sum}_{i=1}^n}{z}_i{h}_i $$

Where:

$$ z=\frac{n+1}{2}-{\lambda}_i $$

And, *n* is the number of individuals in a given population, *λ*_*i*_ denotes the socioeconomic rank of the individual ranging from the richest (*λ*_*i*_ = 1) to the poorest (*λ*_*i*_ = n), *h*_*i*_ is the level of the variable of interest for individual *i*, *b*_*h*_ is the upper bound of the variable of interest, and *a*_*h*_ is the lower bound of the variable of interest. Erreygers concentration index satisfies the four criteria argued by Erreygers to be essential [29, 31] namely, 1) **transfer:** A small transfer of the variable of interest from a richer to a poorer individual is translated into a pro-poor change in the concentration index, 2) **mirror:** The concentration index of the presence of the variable of interest, and the concentration index of the shortfall of the variable of interest should be mirror images of each other, 3) **level independence:** An equal increment of the variable of interest for all individuals does not affect the concentration index, 4) **Cardinal invariance:** A linear transformation of the variable of interest does not affect the value of the index.

#### Health care spending and impoverishment

To assess the impact of OOP (both pure health care and healthcare plus transport) payments on poverty estimates, we first specified a poverty line by adopting the Kenya national poverty line of consumption expenditure of Kenya shillings 1562 (USD 15.62) for rural areas and Kenya shillings 2913 (USD 29.13) for urban areas per individual per month [35]. We then estimated the poverty levels of households and individuals before making healthcare payments (gross of OOP spending) and after making healthcare payments (net of OOP spending [36]. Three measures are presented: (1) the poverty head count represents the fraction of households living below the defined poverty line; (2) the poverty gap represents the aggregate deficit from the poverty line; (3) the normalised poverty gap, which is computed by dividing the estimated poverty gap by the defined poverty line.

#### Examining factors associated with catastrophic costs

We developed a logistic regression model to examine the association of the incidence of catastrophic expenditures and a range of household level and county level covariates. We developed two models, one with catastrophic expenditure due to healthcare costs as the dependent variable, and a second one with catastrophic expenditure due to healthcare and transport costs as the dependent variable.

Covariates were selected based on findings from literature on determinants of catastrophic expenditures [9, 37–40]. Household level covariates included: 1) the age of household head, specified as a categorical variable with 5 categories (less than 25 years, 25–34 years, 35–44 years, 45–55 years and greater than 55 years), 2) the gender of the household head, specified as a binary variable (male or female), 3) the employment status of the household head, specified as a binary variable (employed or unemployed), 4) the socio-economic status of the household, specified as a categorical variable (quintile 1 (poorest), quintile 2, quintile 3, quintile 4, quintile 5 (richest), 5) the household size, specified as a continuous variable, 6) the proportion of household members with some form of health insurance, specified as continuous variable, 7) the presence of at least one household member with a chronic disease (hypertension, diabetes, cancer, arthritis, other), specified as a binary variable (presence or absence of a chronic disease diagnosis), 8) the presence of at least one elderly (greater than 60 years of age) member of a household, specified as a binary variable (presence or absence of an elderly member), 9) the number of healthcare facilities (public, private, faith based) in the county where the household is located, specified as continuous variable, 10) the per capita total healthcare expenditure in the county where the household is located, specified as a continuous variable, 11) the region where the household is located, specified as binary variable (rural or urban), and 12) the status of marginalization of the county where the household is located, specified as binary variable (marginalized or non-marginalized). The marginalization status of the counties is based on the Kenya County Marginalization index, which is a composite index developed by the Kenyan county revenue authority to characterize counties according to the extent to which they are marginalized [41]. The CDI has been developed by combining the poverty status (weight =0.28), education status (weight = 0.28), health status (weight = 0.28) and infrastructure endowment (weight 0.16) of each county to develop a composite index with values ranging from 0 to 1 [41]. Counties with CDI values lower than the national average (0.6) are classified as marginalized while counties with a CDI greater than 0.6 are classified as non-marginalized [41]. Based on this classification, there are 20 marginalized and 27 non marginalized counties [41]. Standard techniques were applied to test for model fit. Data were analyzed using STATA (Version 12).

## Results

### OOP health spending to cover direct healthcare costs and transportation to seek care

Table 2 presents the proportion of households reporting an illness in the 4 weeks preceding the survey.

**Table 2:** Proportion of households reporting illness over a 4 week recall period

	No of Households reporting an illness	Total no of households	% of households reporting an illness (95% CI)
Socio-economic quintiles			
1 (Poorest)	3332	5759	57.86% (56.6–59.1%)
2	3310	5763	57.44% (56.1–58.7%)
3	3374	5763	58.55% (57.3–59.8%)
4	3424	5762	59.42% (58.1–60.7%)
5 (Richest)	3304	5762	57.34% (56.1–58.6%)
Region			
Rural	10,575	17,524	60.35% (59.62–61.07%)
Urban	6169	11,285	54.67% (53.74–55.59%)
Total	16,745	28,810	58.12% (57.55–58.69%)

**Fig. 1:**
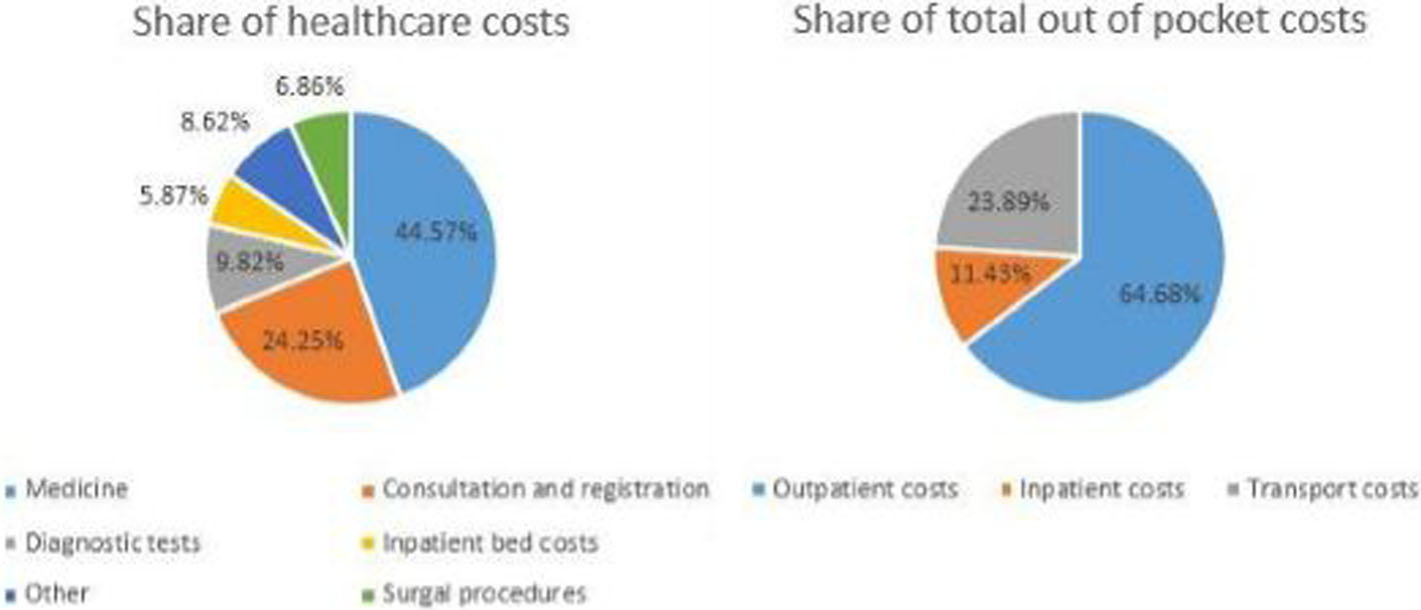
Share of OOP payments to access healthcare services

Table 3 presents estimates of average household OOP spending in a year for all households. Mean annual OOP health spending was KES 5325.12 for outpatient services and KES 941.04 for hospital admissions. Additionally, households on average spent KES 1966.67 on transport to and from a facility to seek outpatient and/or inpatient care. Transport costs therefore equaled almost a third (31.39%) of direct healthcare (inpatient plus outpatient) costs, and comprised 23.89% of total (direct healthcare plus transport) costs incurred to access healthcare (Fig. 1). The proportion of transport costs to total costs incurred to access healthcare was higher among the poor (31.41% in the poorest quintile) compared to the rich (17.50% in the richest quintile). Richer households incurred higher OOP (health care, transport and total) costs to access healthcare services compared to poorer households. Further OOP costs for households residing in urban regions were higher than those residing in rural regions.

**Table 3:** Household mean annual OOP spending (in Kenya shillings) to access healthcare among all households (*n* = 28,810)

	Mean annual outpatient (OP) healthcare costs	Mean annual inpatient (IP) healthcare costs	Mean annual total healthcare (OP + IP) costs	Mean annual transport costs	Mean annual total costs (transport + healthcare costs)
Socio-economic quintiles					
1 (Poorest)	3246.41 (2719.26–3773.57)	348.18 (256.14–440.21)	3594.59 (3055.01–4134.17)	1645.88 (1527.51–1764.24)	5240.47 (4649.58–5831.36)
2	3664.30 (3130.73–4197.88)	606.83 (448.75–764.91)	4271.13 (3703.18–4839.08)	1659.70 (1551.56–1767.84)	5930.83 (5316.14–6545.52)
3	4.,596.04 (4099.75–5092.33)	821.76 (574.91–1068.60)	5417.80 (4833.47–6002.14)	2092.64 (1772.65–2412.62)	7510.44 (6795.31–8225.57)
4	5608.13 (4949.72–6266.54)	1022.51 (706.30–1338.72)	6630.64 (5886.16–7375.11)	2008.35 (1843.95–2172.75)	8638.99 (7791.77–9486.21)
5 (Richest)	9511.66 (8518.25–10,505.06)	1906.08 (1351.27–2460.88)	11,417.73 (10,212.25–12,623.21)	2427.14 (2208.29–2645.99)	13,844.88 (12,511.07–15,178.68)
Region					
Urban	6345.39 (5839.97–6850.81)	1078.77 (842.69–1314.84)	7424.15 (6838.19–8010.11)	1472.21 (1292.60–1651.82)	8896.37 (8235.54–9557.19)
Rural	4667.96 (4298.45–5037.47)	852.34 (675.55–1029.13)	5520.30 (5095.78–5944.82)	2285.42 (2193.06–2377.77)	7805.72 (7330.56–8280.88)
All	5325.12 (5025.47–5624.78)	941.04 (799.21–1082.86)	6266.16 (5920.52–6611.80)	1966.67 (1876.52–2056.83)	8232.83 (7844.81–8620.86)

Direct OOP spending for outpatient services were the greatest driver (64.68%) of total OOP health spending (direct healthcare and transport costs), while payments for medicines comprised the highest proportion (44.57%) of direct healthcare payments (outpatient and inpatient OOP) (Fig. 1).

**Fig. 2:**
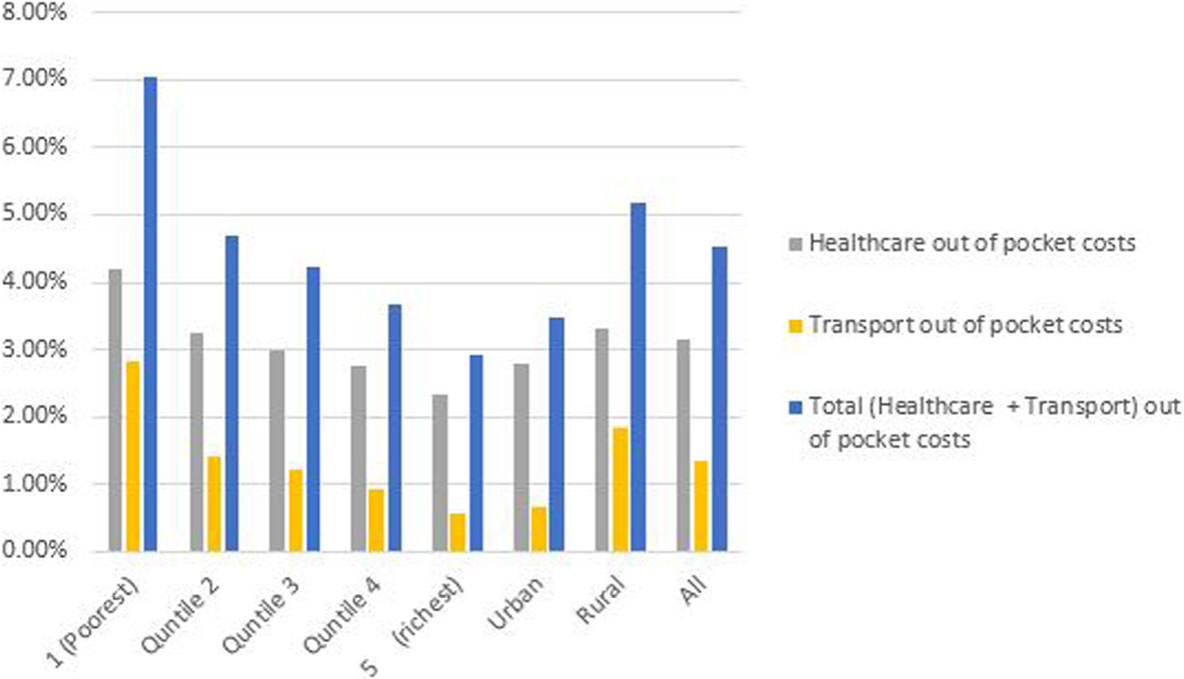
Mean proportion of annual OOP payments to household annual consumption budgets for all households

Figure 2 shows the share of total household expenditure that went towards OOP payments among all households. Overall, OOP payments made directly to healthcare providers to access outpatient and inpatient services, expenditure on transportation to access care, and total OOP payments (direct payments to healthcare providers plus return trip costs) represented 3.14, 1.37 and 4.51% of a household’s total annual consumption expenditures respectively. The poorest households and those living in rural regions spent the largest share of their annual budgets on healthcare compared to richer households and those living in urban areas respectively. For instance, the share of household annual budgets that was spent by the poorest household (quintile 1) to access healthcare (healthcare and transport costs) was 3 times (7.04%) that of the richest (quintile 5) households (2.34%). For rural households, their budget share for total OOP (healthcare and transport) costs was 5.16% compared to 3.47% for urban households.

### Impoverishing effects of Catastrophic Expenditure

Table 4 outlines the estimates of the incidence and intensity of catastrophic health payments. Overall, the incidence of catastrophe was 4.52% when only healthcare OOP payments made directly to healthcare providers were considered, and rose to 6.57% when both direct healthcare OOP payments and transport costs were considered. Richer households had a significantly lower incidence of catastrophe compared to poorer households. For instance while 1.29 and 2.40% of households in the highest socio-economic quintile incurred catastrophic costs due to healthcare and healthcare plus transport costs respectively, the incidence of catastrophe in households in the poorest socio-economic quintile was 9.92% (for healthcare OOP costs) and 15.68% (for healthcare plus transport OOP costs). Further, the incidence of catastrophe was significantly higher among households in rural compared to households in urban regions of the country.

**Table 4:** Incidence and intensity of catastrophic health expenditure

	Total healthcare costs	Transport	Total (Healthcare plus transport)
Head count			
Socio-economic status			
1 (poorest)	9.92% (8.38–11.47%)	5.91% (4.51–7.32%)	15.68% (13.81–17.54%)
2	4.56% (3.81–5.31%)	1.12% (0.76–1.54%)	7.18% (6.24–8.11%)
3	4.47% (3.67–5.28%)	0.76% (0.04–1.03%)	5.81% (4.94–6.69%)
4	2.75% (2.19–3.32%)	0.25% (0.10–0.41%)	3.61% (2.95–4.27%)
5 (Richest)	1.92% (1.39–2.47%)	0.00% (0.00–0.02%)	2.40% (1.81–2.99%)
Regions			
Urban	3.59% (2.92–4.25%)	0.38% (0.16–0.59%)	4.30% (3.58–5.02%)
Rural	5.01% (4.47–5.53%)	2.02% (1.59–2.46%)	7.78% (7.06–8.50%)
Weighted total head count	4.52% (4.10–4.93%)	1.46% (1.15–1.76%)	6.58% (6.03–7.12)
Overshoot			
Socio-economic status			
1 (poorest)	11.22% (7.63–14.81%)	5.74% (2.73–8.76%)	19.44% (13.87–25.01%)
2	4.25% (2.72–5.78%)	0.41% (0.26–0.56%)	5.64% (3.98–7.31%)
3	2.37% (1.62–3.13%)	0.27% (0.14–0.39%)	3.30% (2.40–4.20%)
4	1.73% (1.01–2.39%)	0.07% (0.00–0.13%)	2.11% (1.38–2.84%)
5 (Richest)			
Regions			
Urban	2.16% (1.45–2.86%)	0.10% (0.02–0.19%)	2.56% (1.81–3.33%)
Rural	4.58% (3.48–5.68%)	1.68% (0.86–2.49%)	7.39% (5.73–9.05%)
Weighted overshoot (OW)	3.75% (2.98–4.51%)	1.14% (0.60–1.67%)	5.73% (4.60–6.86%)
Concentration index, C_E	−0.30 (−0.35–−0.26)	−0.69 (−0.77–−0.61)	−0.35 (−0.39–−0.32)
Concentration Index, C_O	−0.53 (−0.71–−0.34)	−0.80 (−1.11–−0.49)	−0.57 (−0.72–−0.41)

The mean positive overshoot was 3.75% when only direct OOP paid to healthcare providers are considered and 5.73% if both direct OOP costs paid to healthcare providers and transport costs are considered. The mean positive overshoot represents the extent to which OOP payments exceed the defined catastrophic threshold (40% of non-food budget share). The concentration indices are negative for both catastrophic expenditures and overshoot, showing that both the incidence and intensity of catastrophe are concentrated among the poor.

**Fig. 3:**
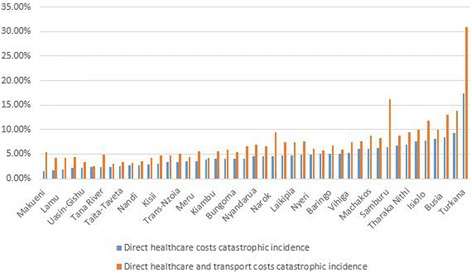
Incidence of catastrophic head count by county

Figure 3 shows that the incidence of catastrophe due to OOP costs incurred to access healthcare services varied across the 47 Kenyan counties. Of the 47 counties, level of catastrophic expenditure (for both direct healthcare costs, and direct healthcare plus transport costs) was higher than the national average in 21 counties. When healthcare OOP costs are considered, Turkana county had the highest (17.36%) incidence, while Makueni county had the lowest (1.57%). When both healthcare and transport OOP costs were considered, once again Turkana county had the highest (30.91%) incidence, while Nakuru county had the lowest (2.59%).

Table 5 outlines the poverty head count before and after accounting for OOP payments. The results reveal that while 66.60% of individuals were already living below the national poverty line before making any payments to access healthcare services, this fraction increased by 1.17 percentage points and 1.60 percentage points after accounting for healthcare OOP and healthcare plus transport OOP respectively. This implies that an estimated 453,470 individuals are pushed into poverty annually as a result of healthcare OOP. When both direct payments made to healthcare providers and transport OOP costs are considered, an estimated 619,541 individuals are pushed into poverty annually. The poverty gap, which represents the average deficit to reach the poverty line in the population was KES 16,245.71 before accounting for any healthcare OOP payments. This gap increased to KES 16,989.57 and KES 17,239.39 when healthcare OOP and healthcare plus transport OOP were accounted for respectively.

**Table 5:** Poverty head count before and after OOP payments

	Gross of total (health + transport) payments	Net of health payments	Net of transport payments	Net of (health and transport) payments
Poverty headcount	66.60% (64.95–68. 26%)	67.78% (66.15–69. 41%)	67.02% (65.37–68.67%)	68.21% (66.58–69.84%)
Poverty gap	16,245.71 (15,528.48–16,962.94)	16,989.57 (16,263.97–17,715.16)	16,465.69 (15,745.25–17,186.13)	17,239.39 (16,510.55–17,968.23)
Normalized poverty gap	30.26% (29.10–31.41%)	31.65% (30.50–32.81%)	30.72% (29.56–31.89%)	32.19% (31.02–33.36%)
Mean positive poverty gap	24391.74 (23656.09–25127.39)	25066.82 (24324.57–25809.07)	24568.1 (23833.54–25302.67)	25275.11 (24536.74–26013.47)
Normalized mean positive poverty gap	45.43% (44.55–46.30%)	46.70% (45.84–47.57%)	45.84% (44.96–46.71%)	47.19% (46.32–48.06%)

**Fig. 4:**
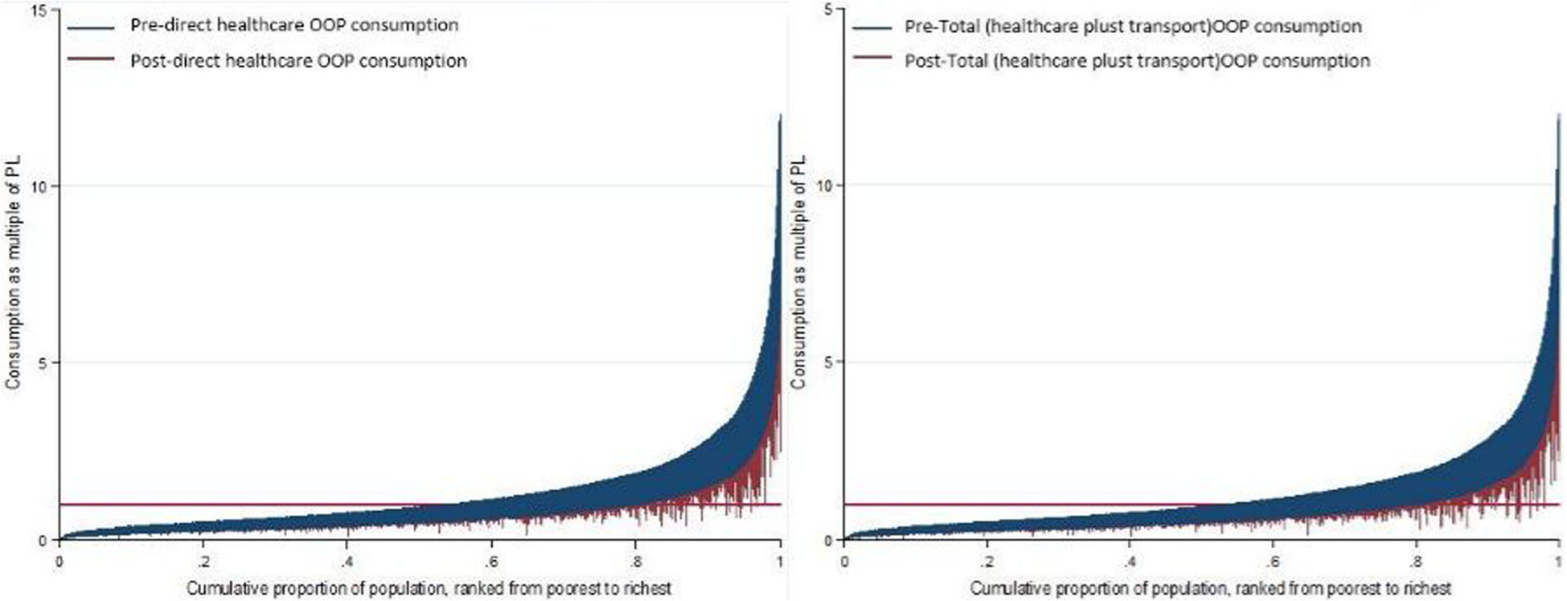
Effect of OOP Payments on Household Consumption

To graphically illustrate the impoverishing effects of OOP payments, Fig. 4 shows Pen’s Parade for household consumption gross of OOP made directly to healthcare providers and for transport OOP. Household consumption is expressed here as multiples of the Kenyan national poverty line (PL). For each household, the vertical bar, or “paint drip,” shows the extent to which the subtraction of OOP payments reduces consumption. If a bar crosses the poverty line, it means that this household is potentially pushed into poverty due to OOP payments to access healthcare. The graph shows that, a significant portion of the population is already poor and that OOP payments push them further into poverty. OOP payments are largest in the highest (richest) quintile, while households in the 4^th^ quintile are the ones that are pushed into poverty by OOP payments to access healthcare.

### Factors associated with the incidence of catastrophic expenditure

Table 6 presents the results of logistic regression models that examine the relationship between the incidence of catastrophic health expenditure at the household level and a range of individual, household and county level co-variates. We developed two models, the first with a binary variable measuring whether the household experienced catastrophic expenditure due to direct OOP made to healthcare providers as the dependent variable and the second with a binary variable indicating whether direct healthcare plus transport OOP were catastrophic for a household as the dependent variable.

**Table 6:** Logistic model estimation for likelihood of incurring Catastrophic Health Expenditure (CHE) [incurred CHE = 1, 0 = otherwise]

	Catastrophic expenditure due to healthcare OOP costs as the dependent variable			Catastrophic expenditure due to healthcare and transport OOP costs as the dependent variable		
Independent variables	Odds Ratio (95% CI)	Standard error	*P* Value	Odds Ratio (95% CI)	Standard error	*P* Value
Age of household head (reference = less than 25 years)						
25 years–34 years	1.30 (1.05–1.62)	0.15	0.02	1.20 (1.00–1.45)	0.12	0.05
35 years–44 years	1.50 (1.08–2.08)	0.25	0.02	1.44 (1.09–1.91)	0.21	0.01
45 years–54 years	1.88 (1.31–2.71)	0.35	0.00	1.60 (1.17–2.18)	0.25	0.00
over 55 years	1.72 (1.15–2.58)	0.36	0.01	1.61 (1.14–2.28)	0.29	0.00
Gender of household head (reference = female)	1.01 (0.85–1.19)	0.08	0.94	1.08 (0.94–1.25)	0.08	0.28
Employment status of household head (reference = employed)	1.75 (1.42–2.16)	0.19	0.00	1.61 (1.35–1.92)	0.15	0.00
Socio-economic status (reference = quintile 5 (richest))						
Quintile 4	1.57 (1.09–2.27)	0.29	0.02	1.56 (1.15–2.13)	0.25	0.00
Quintile 3	2.65 (1.83–3.84)	0.50	0.00	2.57 (1.87–3.53)	0.42	0.00
Quintile 2	2.79 (1.97–3.95)	0.49	0.00	3.22 (2.39–4.35)	0.49	0.00
Quintile 1 (Poorest)	5.61 (3.83–8.22)	1.09	0.00	6.81 (4.99–9.28)	1.08	0.00
Household size	(1.00–1.11)	0.03	0.04	1.04 (1.00–1.08)	0.02	0.04
Proportion of household members with health insurance cover	1.44 (1.00–2.08)	0.27	0.05	1.25 (0.91–1.72)	0.20	0.17
Chronic disease diagnosis in the household (reference = no chronic disease)	2.24 (1.87–2.68)	0.20	0.00	2.03 (1.75–2.34)	0.15	0.00
Presence of at least one elderly(>60 years) member in the household (reference = no elderly member)	1.31 (1.02–1.68)	0.17	0.04	1.23 (1.01–1.51)	0.13	0.04
Number of healthcare facilities in the county	1.00 (1.00–1.00)	0.00	0.12	1.00 (1.00–1.00)	0.00	0.39
Per capita total health expenditure in the county	1.00 (1.00–1.02)	0.01	0.65	1.01 (1.00–1.02)	0.01	0.14
Region (reference = rural region)	0.89 (0.73–1.08)	0.09	0.23	0.72 (0.61–0.85)	0.06	0.00
Marginalization status of the county (reference = non marginalized)	1.38 (1.14–1.67)	0.13	0.00	1.51 (1.29–1.78)	0.13	0.00

Households had increased odds of incurring catastrophic expenditures due to healthcare costs if their household head was older. For instance, households whose heads were over 55 years of age had a 72% increased odds incurring catastrophic expenditures due to direct healthcare costs compared to households whose heads were below 25 years of age (Odds ratio OR =1.72, 95% CI 1.15–2.58). A household with an unemployed head had 75% more odds of incurring catastrophic expenditure due to direct healthcare costs compared to a household with an employed head (OR =1.75, 95% CI 1.42–2.16). Low social economic status also increased the odds of a household incurring catastrophic expenditure. For example, households in the poorest quintile had 5.61 times more odds of incurring catastrophic expenditure due to direct healthcare costs compared to the households in the richest quintile (OR = 5.61, 95% CI 3.83–8.22). Other variables that increased the odds of incurring catastrophic costs due to direct healthcare costs include household with a larger number of household members (OR =1.05, 95% CI 1.00–1.11), households with a household member with a chronic disease (OR = 2.24, 95% CI 1.87–2.68) or an elderly household member (OR = 1.31, 95% CI 1.02–1.68), and households that were located in a marginalized county (OR =1.38, 95% CI 1.14–1.67) (Table 6). The same variables were significantly associated with an increased odds of the incidence of CE due to direct healthcare plus transport costs (Table 6).

## Discussion

This study presents a detailed analysis of catastrophic health spending in Kenya using the most recent nationally representative household survey. Direct comparison of our findings with those from other settings is limited by differences in methodological choices such as how health expenditure was measured and choice of the threshold for catastrophic health expenditures. This not-withstanding, our findings appear to be comparable to those reported elsewhere in the Sub-Saharan Africa region [42, 43]. For example, using the 40% of non-food expenditure threshold, a recent study in Zambia reported an incidence of catastrophic expenditure to direct healthcare costs of 4.00%, which increased to 9.30% when transport costs were included [43].

When compared to similar analysis in Kenya from previous periods, our results show that the country is moving in the right direction. Compared to 2007, when 14.8% of Kenyan households incurred catastrophic expenditures due to direct costs of healthcare [20], only 4.52% of households incurred catastrophic expenditure due to direct costs healthcare in 2013. As a result, while close to 1.5 million Kenyans were pushed into poverty due to OOP direct costs of healthcare in 2007 [20], this number reduced to 453,470 in 2013. While this analysis does not explore the causes for this reduction, we suspect that this could be in part the result of a recent government policy to abolish user fees at public primary healthcare facilities (health centers and dispensaries) in 2013 [16]. In the context of the finding that outpatient care contributes the greatest proportion (64.68%) of direct healthcare costs, and the observation that the greatest proportion (40.10%) of outpatient visits occur in public primary healthcare facilities (health centers and dispensaries) [17], the user fee removal policy has the potential for significantly increasing financing risk protection among the Kenyan population.

Further, the fact that the level of catastrophic expenditure due to direct healthcare costs is still high reinforces the observation made in other countries that user fee removal is not enough. For example studies from Uganda, Bukina Faso and Zambia reported that user fee removal did not reduce, and sometimes even increased OOP payments to access healthcare services [43–46]. In the three countries, this was attributed to poor quality of care in healthcare facilities, such as the unavailability of essential medicines and supplies [43–46]. It is likely that similar reasons explain the persistence of catastrophic expenditures due to direct healthcare costs in Kenya. Indeed data from the KHHES 2013 shows that lack of trained personnel and medicines were the leading reasons for patients bypassing facilities closer to them when seeking care (data not shown). Further, data from the KHHEUS 2013 shows that approximately 39.90% of those who sought care in public primary healthcare facilities did incur some direct OOP expenditure (data not shown). This implies poor adherence to the policy, at least at the time of the survey. This finding adds to the evidence that financial risk protection is closely related to the quality of care offered in healthcare facilities and the fidelity of implementation of free care policies [47].

Our results highlight the financial burden that paying for transportation to healthcare facilities poses for Kenyan households and especially the poor. It is worth noting that assessments of catastrophic health spending in LMICs typically focus on direct healthcare payments, and customarily ignore other indirect costs such as the cost of transportation to the health facility. The seeming vulnerability among the poor is not only due to the poor having lower incomes, but also because a significant proportion of the poor live in rural and marginalized regions of the country, where access to healthcare facilities is limited because facilities are few and far between. Our study thus adds to the growing evidence that transport costs comprise a significant proportion of OOP healthcare costs. For example, findings from Zambia report that transport costs comprised 73.00% of OOP costs incurred access healthcare services in Zambia [43]. Transport has been identified as a significant barrier to access in a number of settings [48–50].

Our findings also reinforces evidence from other settings that outpatient care and costs of medicines are the greatest cost drivers of direct OOP costs paid to healthcare facilities [43, 51]. This finding is important given that often health financing schemes, and specifically social health insurance schemes in LMICs do not adequately and or/explicitly cover the cost of medicines and outpatient care [52]. For example, at the time of collecting data for the KHHEUS 2013, the Kenyan NHIF provided an inpatient care only benefit package and did not explicitly include medicines in the package. While the NHIF expanded its benefit package to include outpatient care in 2015, essential medicines are still not explicitly included.

Our study offers some insight into who amongst the Kenyan population are most vulnerable to catastrophic expenditures. Households that are larger, poorer, have an unemployed head, have a member with a chronic ailment such as diabetes of hypertension, have an elderly member, or live in marginalized regions of the country have an increased odds of incurring catastrophic expenditures. These findings are consistent with evidence from other settings on the determinants of the incidence of catastrophic health expenditures. For example, the presence of a household member with a chronic illness, or the unemployment of the household head were found to increase the odds of a household incurring catastrophic health expenditures in Nepal [53], China [37], Kenya [54], and Ghana [51]. Larger household sizes were also found to increase odd of incurring catastrophic health expenditures in Ghana [51] and Iran [55].

Our findings on the influence of health insurance on health spending add to the mixed findings from the literature on this topic. While some studies have found that having health insurance reduces the odds of incurring catastrophic health expenditures [9, 51, 56, 57], findings in other settings have shown that the expansion of health insurance does not necessarily increase financial risk protection in the population [52, 58, 59]. Despite the increase in health insurance coverage from 10.00 to 17.10% between 2007 and 2013 [17] in Kenya, our analysis does not find health insurance to be protective of catastrophic expenditures. This could be explained by a number of reasons. First, as mentioned previously, at the time of collecting data for the KHHEUS, the NHIF benefit package did not include outpatient services. As we have shown, outpatient costs are significant cost drivers of OOP in Kenya. Second, the NHIF in Kenya majorly contracts hospitals to provide healthcare services to its members, while the majority of outpatient visits in Kenya are in primary healthcare facilities [17]. Third, access to care by NHIF members is severely constrained; the network of facilities contracted by NHIF to provide services to its members is small (approximately 1400 facilities out of approximately 10,000 healthcare facilities in Kenya), with most of these being in Urban areas [60]. The majority of Kenyan live in rural regions of the country.

### Implications for policy

Our analysis has a number of implications for policy in Kenya and similar settings. First, the design of health financing mechanisms should prioritize the cost drivers of OOP spending. For example, social health insurance schemes, such as the NHIF in Kenya, should explicitly include essential medicines in their benefit packages. Further, in addition to policies to remove user fees paid directly to health facilities, policy makers should explore policies to reduce the burden of transport costs especially among the poor, and the vulnerable. For example, while the government of Kenya has implemented a free maternity healthcare programme and introduced a health insurance subsidy for the poor programme [60], they should explore introducing transport vouchers to further reduce the financial burden of accessing care.

Second, policies aimed at providing financial risk protection should prioritize vulnerable groups in the population. For instance, interventions for prevalent chronic diseases such as diabetes and hypertension should be included in benefit packages of UHC schemes. Further, in extending coverage, special priority should be given to the poor, the elderly and those living in rural and marginalized regions of the country. While the Kenyan government has introduced an insurance subsidy programme for the poor and the elderly, these remain pilots funded by donors and therefore have very limited coverage [60]. The Kenyan government should allocate a budget for scaling up coverage to these vulnerable segments of the population.

Third, efforts at reducing financial barriers to access can only succeed if accompanied by efforts to remove supply side bottlenecks. Specifically, the Kenyan government should invest in increasing geographical access to healthcare facilities among the population, especially those in the rural and marginalized regions of the country. The NHIF should also scale up the number of facilities it contracts to provide healthcare services to its members, and specifically focusing on increasing the numbers of primary healthcare facilities, and the network of contracted facilities in rural and marginalized areas. The Kenyan government should also prioritize implementing measures to improve the quality of care in health facilities. The government should strengthen the supply chain and availability of essential medicines and supplies in public healthcare facilities, while the NHIF should strengthen its purchasing function to ensure that essential medicines and supplies are available in both the public and private facilities that it contracts to provide services for its members.

### Study limitations

The KHHEUS was conducted in 2013, which makes it outdated. However, KHHEUS is the only data source that provides detailed information on health consumption patterns at the household-level in Kenya, and the 2013 round is the most recent round. Further, as has been observed by others, surveys such as the KHHEUS rely on self-reported data on healthcare use, which is susceptible recall bias [61].

## Conclusion

KHHEUS 2013 offers us the most recent estimate of catastrophic health spending in Kenya. While there is an observed decline in both the incidence of catastrophic health spending and its impoverishing effects, the levels are still unacceptably high especially among the poor. There are signs that recent government of Kenya reforms, specifically the user fee removal policy might have contributed to increased financial risk protection among the poor. The fact however that CHE persist in the Kenyan population means that the Kenyan government has to do more to not only ensure that prepayment mechanisms are scaled up and adhered to, but that supply side bottlenecks such as quality of care and geographical access to facilities are improved. It is also clear from our analysis that transport costs comprise a significant portion of OOP and should be given policy priority by policy makers, especially among the poor.

## Abbreviations

KHHEUS: Kenya Household Health Expenditure and Utilization Survey
MOH: Ministry of Health
NHIF: National Hospital Insurance Fund
OOP: Out of pocket
THE: Total health expenditure
